# Genome-wide association study, haplotype analysis, and genomic prediction reveal the genetic basis of yield-related traits in soybean (*Glycine max* L.)

**DOI:** 10.3389/fgene.2022.953833

**Published:** 2022-08-17

**Authors:** Javaid Akhter Bhat, Kehinde Adewole Adeboye, Showkat Ahmad Ganie, Rutwik Barmukh, Dezhou Hu, Rajeev K. Varshney, Deyue Yu

**Affiliations:** ^1^ Soybean Research Institution, National Center for Soybean Improvement, State Key Laboratory of Crop Genetics and Germplasm Enhancement, Nanjing Agricultural University, Nanjing, China; ^2^ International Genome Center, Jiangsu University, Zhenjiang, China; ^3^ Department of Agricultural Technology, Ekiti State Polytechnic, Isan, Nigeria; ^4^ Plant Molecular Science and Centre of Systems and Synthetic Biology, Department of Biological Sciences, Royal Holloway University of London, Surrey, United Kingdom; ^5^ Center of Excellence in Genomics & Systems Biology, International Crops Research Institute for the Semi-Arid Tropics (ICRISAT), Hyderabad, India; ^6^ Murdoch’s Centre for Crop & Food Innovation, State Agricultural Biotechnology Centre, Food Futures Institute, Murdoch University, Perth, WA, Australia

**Keywords:** haplotype-based breeding, GWAS, legumes, seed yield, candidate genes, crop improvement

## Abstract

Identifying the genetic components underlying yield-related traits in soybean is crucial for improving its production and productivity. Here, 211 soybean genotypes were evaluated across six environments for four yield-related traits, including seed yield per plant (SYP), number of pods per plant number of seeds per plant and 100-seed weight (HSW). Genome-wide association study (GWAS) and genomic prediction (GP) analyses were performed using 12,617 single nucleotide polymorphism markers from NJAU 355K SoySNP Array. A total of 57 SNPs were significantly associated with four traits across six environments and a combined environment using five Genome-wide association study models. Out of these, six significant SNPs were consistently identified in more than three environments using multiple GWAS models. The genomic regions (±670 kb) flanking these six consistent SNPs were considered stable QTL regions. Gene annotation and in silico expression analysis revealed 15 putative genes underlying the stable QTLs that might regulate soybean yield. Haplotype analysis using six significant SNPs revealed various allelic combinations regulating diverse phenotypes for the studied traits. Furthermore, the GP analysis revealed that accurate breeding values for the studied soybean traits is attainable at an earlier generation. Our study paved the way for increasing soybean yield performance within a short breeding cycle.

## Introduction

Soybean (*Glycine* max L. Merr.) is one of the most important food legume crops cultivated globally (Hina et al., 2020). Many countries including China are highly dependent on soybean imports to fulfil their domestic needs (Liu et al., 2018; Karikari et al., 2019). Improving soybean yield has been a primary objective of breeders from China and several other countries across the world (Karikari et al., 2019). Seed yield of soybean is governed by yield-related traits such as the number of pods per plant, number of seeds per plant, and seed weight (Bianchi et al., 2020). However, yield and its component traits are complex, being controlled by multiple genes and are considerably affected by the environment (Liu et al., 2011).

Many studies have revealed the genetic basis of yield-related traits in staple crops such as maize (Badu-Apraku et al., 2020; Yang et al., 2020), rice (Yue et al., 2015; Adeboye K. A. et al., 2021), and soybean (Karikari et al., 2019; Hu et al., 2020) by using bi-parental mapping populations. To date, several hundred QTLs regulating yield and yield-related traits have been mapped across the soybean genome, and many QTLs were consistently identified in different populations (http://www.soybase.org). All these studies have validated the role of linkage mapping as an efficient approach to dissecting the genetic basis of quantitative traits (Karikari et al., 2019; Hina et al., 2020). However, a major limitation of the linkage mapping approach is its dependence on limited diversity existing within segregating populations derived from two contrasting parents. By contrast, GWAS has emerged as an alternative approach, which is more efficient in the identification of QTLs regulating quantitative traits by utilizing natural diversity existing within crop germplasm and the use of high-density genetic markers (Zargar et al., 2015; Yu et al., 2019). Importantly, GWAS has a higher potential to identify candidate genes regulating the trait of interest because of a reduced level of genomic linkage disequilibrium (LD) (Alqudah et al., 2020).

Advances in next-generation sequencing technologies have enabled a wider availability of high-throughput sequencing and genotyping platforms (Bhat et al., 2020; Sahu et al., 2020). As a result, genomics-assisted breeding (GAB) has emerged as the method of choice for crop improvement in plant breeding programs (Varshney et al., 2021). Although, both linkage mapping and GWAS approaches are being successfully used for the identification of QTLs/candidate genes in crop plants, limited allelic diversity and genomic resolution associated with linkage mapping are addressed by GWAS in the gene identification process (Brachi et al., 2011). GWAS has been efficiently used to identify the QTLs/genes underlying various yield-related traits in soybeans such as seed protein and oil content (Zhang et al., 2019), agronomy (Zatybekov et al., 2017), salt tolerance (Zeng et al., 2017), and yield-related traits (Hu et al., 2020). Another genomic-based plant breeding approach is the genomic prediction (GP). Here, phenotypic traits or performance of an individual is predicted based on genomic data. GP is currently being used in multiple plant species to estimate the genetic values (genotypic estimated breeding values (GEBVs)) of the individual genotypes based on the genome-wide genotypic data without the need for phenotypic data (Habier et al., 2007; Bhat et al., 2016; Crossa et al., 2017). The GP allows for the captures of QTLs with minor effects since the model is based on the genome-wide marker data rather than few markers as in the marker-assisted selection model. Thus, it has a great potential for improving the genetic gain associated with yield and other complex traits within a limited time frame in different crop plants (Crossa et al., 2017; Voss-Fels et al., 2019; Lebedev et al., 2020). Moreover, GP has been used in soybean for improving different traits such as cyst nematode infestation (Ravelombola et al., 2020), disease resistance (Rolling et al., 2020), agronomic traits (Beche et al., 2021), and seed yield (Mendonça et al., 2020). The results of these studies have demonstrated the potential of GP for improving complex traits in soybean. Furthermore, advanced sequencing technologies are providing high accuracy in gene and haplotype mining in crop germplasm (Bevan et al., 2017; Bhat et al., 2021).

The present study analyzes the genetic basis of yield-related traits in summer planting soybean genotypes grown in soybean growing areas of China. We evaluated 211 diverse soybean genotypes across six environments for four yield-related traits, including seed yield per plant (SYP), number of pods per plant (PPP), number of seeds per plant (SPP), and 100-seed weight (HSW). Based on the phenotypic performance, genome-wide association analysis was conducted to identify QTLs associated with the studied traits using five different models. The genes underlying the identified QTLs were validated based on RNA-seq data for soybean tissues. Furthermore, superior haplotypes and alleles were identified within the genomic regions associated with the studied traits. Also, genetic values of individual genotypes were estimated based on the studied yield-related traits to facilitate the selection of soybean for improved yield performance.

## Materials and methods

### Plant materials and field experiment

The GWAS panel of soybean used in the current study consists of 211 diverse genotypes; which include 201 genotypes originating from 25 provinces of China that represents all three ecological habitats of China (Zhang S. et al., 2021) and ten genotypes from the United States, Japan, and Brazil (Zhang et al., 2015). This soybean germplasm was evaluated at three different locations in China viz., Nanjing, Nantong, and Yangzhou, for two consecutive years. This makes a total of six different environments viz., E1 and E2 (Nanjing); E3 and E4 (Nantong); and E5 and E6 (Yangzhou). The study location was previously described by Bhat et al. (2022). Nanjing (32°12′ N, 118°37′ E) has north subtropical humid climate. It receives an average rainfall of 1,106.5 mm, 76% average relative humidity, and 15.4°C average temperature. Nantong (31°58′ N, 120°53′ E) is located at the lower reaches of Yangtze River in the alluvial plain with mild marine climate, and possesses an average temperature and precipitation of 15.1°C and ∼1,040 mm, respectively. Yangzhou (32°23′ N, 119°25′ E) is located in the southern end of Yangtze Huaihe plain, and receives an average precipitation and temperature of 1,020 mm and 14.8°C, respectively. In each environment, all the 211 diverse soybean genotypes were planted in a randomized complete block design with three replications. Each genotype was planted in a plot of three rows with row-length and spacing of 200 and 50 cm, respectively. Normal agronomic practices were followed for the cultivation of soybean germplasm at each location, as previously described by Zhang S. et al. (2021).

### Phenotypic data collection and analysis

At maturity, five consecutive plants of each genotype were selected from the middle of each plot for data collection. Phenotypic data were recorded for four yield-related traits including seed yield per plant (SYP), number of pods per plant (PPP), number of seeds per plant (SPP), and 100-seed weight (HSW). The phenotypic data were subjected to analysis of variance with the mixed linear model using *lme4* – an r-package implemented in PBTools v1.4 (IRRI, 2014). BLUPs were generated for GWAS by setting the genotype as random. Pearson correlation coefficient between traits was determined at *p < 0.05* and visualized using MVApp (Julkowska et al., 2019).

### Genotyping, linkage disequilibrium, and genome-wide association study

NJAU 355 K Soy SNP Array previously developed and described by Wang et al. (2016) was used in this study. Quality control analysis was performed using PLINK v1.07 (Purcell et al., 2007) with the following criteria: missing genotype and individual at 0.1; minor allele frequency (MAF) at 0.01, and Hardy-Weinberg exact test at 0.000001. For the genome-wide LD analysis, pairwise squared allele-frequency correlations (*r* ^2^) between SNP markers with known genomic positions were calculated using Trait Analysis by Association, Evolution, and Linkage (TASSEL) v5.72 (Bradbury et al., 2007) with 100 sliding window sizes. The expected values of *r* ^2^ under drift equilibrium were calculated according to Hill and Weir (1988) and plotted against physical distance (Kbp). The LD decay curve line was fitted on the scatterplot using the smoothing spline regression line at the genome level following the procedure of Remington et al. (2001) in the *R* environment.

The GWAS was performed using the following five models:

1) General linear model (GLM) with principal component analysis (PCA) to reduce false positive association due to population structure (Price et al., 2006) based on the equation as follows: 
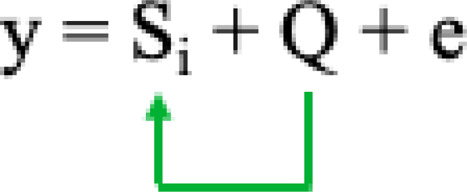

Where Si = testing marker, and Q = Population structure.

2) The compressed mixed linear model (CMLM) (Zhang et al., 2010), has increased statistical power for marker-trait association detection relative to other methods. CMLM method groups individuals into clusters, and random effects are fitted as genetic values of clusters in a mathematical model: 
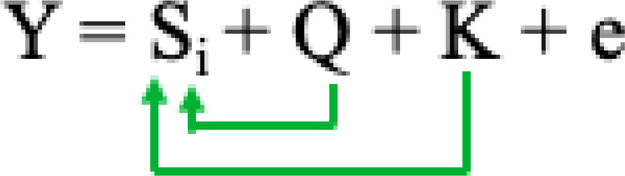

Where Si = testing marker, Q = Population structure, and K = Kinship by group.

3) The multiple-locus mixed linear model (MLMM) (Segura et al., 2012) incorporates the kinship matrix and Pseudo Quantitative Trait Nucleotide (QTN) to control false discovery rate (FDR) based on the model equation: 
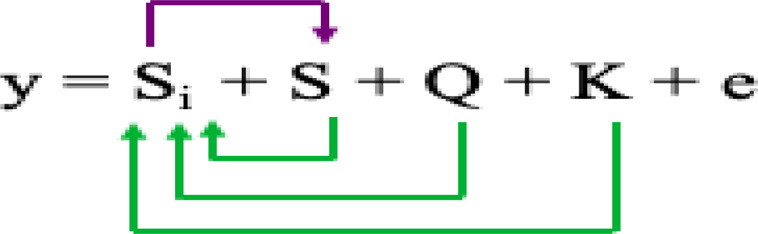

Where Si = testing marker, Q = Population structure, K = Kinship of individuals, and S = Pseudo QTN.

4) The fixed and random model circulating probability unification (FarmCPU) (Liu et al., 2016) iteratively uses the Fixed Effect Model (FEM) and a Random Effect Model (REM) as shown in the model equation: 
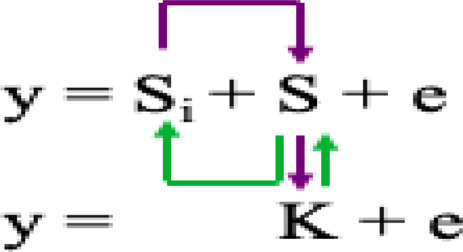

Where K = kinship derived from only the associated markers or Pseudo QTN (S) using maximum likelihood method.

5) Bayesian-information and linkage-disequilibrium iteratively nested keyway (BLINK) (Huang et al., 2019) is an improved version of FarmCPU model and expressed as below: 
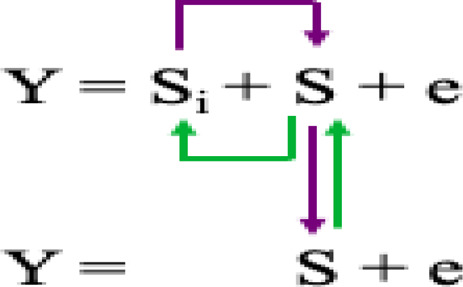

S = pseudo QTNs that are not in LD with each other selected in two FEMs and one filtering process and optimization using Bayesian information criterion (BIC).

Among the five GWAS models used, GLM and CMLM are single locus models; whereas, MLMM, FarmCPU, and BLINK are the multi-locus models. All of the models were implemented using GAPIT v3 package (Lipka et al., 2012) in an *R* environment. To correct for the population structure, PCA was employed using BIC to estimate the optimal numbers of PCA (Schwarz, 1978). The population structure was visualized based on soybean ecological regions in China using the “*ggbiplot*” package in the *R* environment. Significant SNP associations were determined according to the inherently included method in GAPIT v3 (Lipka et al., 2012) as the negative logarithm of *p*-value (where *p*-value = 0.01/number of markers, and 0.01 represents MAF cut-off). Furthermore, only SNPs that were commonly detected across a minimum of two models were considered for further analysis.

### Prediction of candidate genes

The genes lying upstream and downstream of each stable SNP (within the LD decay distance of the studied population) were obtained from the freely available online RNA-seq data for different soybean tissues on SoyBase website (https://www.soybase.org). Functional descriptions of these genes were also obtained, manually screened, and presented in a heatmap.

### Allele-effect and haplotype analysis

Effects of alleles underlying the significant stable SNP markers were analyzed as previously described by Su et al. (2019) and Alemu et al. (2021). Genotypes were grouped into independent groups according to their specific SNP alleles, and means were compared using Turkey’s HSD test.

Haplotype analysis was conducted using PLINK v1.07 (Purcell et al., 2007). The stable SNP markers (identified in two or more two environments) were considered reference markers for building haplotype blocks/loci. Besides, all markers that were in close association with the reference SNP markers within the estimated LD decay distance of the studied population formed a haplotype block/locus. Effects of haplotype alleles on the studied traits were tested across all the six environments using the conditional haplotype testing command (*--chap*). Also, the contribution of each haplotype to the observed phenotypic variance across the environments was estimated using the *"--hap-assoc*” command (Purcell et al., 2007) and visualized in Microsoft Excel.

### Genomic prediction

The genomic prediction was explored for each trait in individual and combined environments using Genomic best linear unbiased prediction (gBLUP) and the ridge regression best linear unbiased prediction (rrBLUP) based on the mixed-model:

y = Xβ + Zμ + ε

where β and μ represent the vectors of fixed and random effects, respectively, and ε is the residual error.

The gBLUP was implemented in TASSEL v5.72 (Bradbury et al., 2007). Cross-validation was achieved in five-folds with 20 iterations to test the genomic prediction accuracy and to avoid overfitting of the model. The rrBLUP on the other hand was implemented using the “rrBLUP” package (Endelman, 2011; Endelman and Jannink, 2012) in the R environment. To validate the genomic prediction accuracy, the dataset was randomly divided into training and testing sets at 80 and 20% respectively. To manage the challenges of overfitting, the cross-validation was conducted in five hundred cycles of iterations. The predictive ability was estimated as the Pearson’s correlation coefficient between the observed and predicted phenotypic values of the test set based on the effect estimates of genotypes in the training set.

## Results

### Phenotypic analysis

Analysis of variance for the four yield-related traits evaluated across 211 soybean genotypes is summarized in Table 1. A highly significant variation (*p < 0.0001*) was observed for the genotype, environment, and G × E interaction in all the studied traits. However, the estimates of variance components varied across different traits (Table 1). For all the studied traits, the genotype component accounted for the highest proportion of the observed variations. Moreover, medium to high broad-sense heritability (*h* ^2^) was observed, ranging from 0.61 (SYP) to 0.99 (HSW) in individual environments (Supplementary Table S1) and from 0.80 (SYP) to 0.99 (HSW) in the combined environment (Table 1).

**TABLE 1:** Combined analysis of variance and broad-sense heritability for four yield and yield-related traits.

Traits	PPP	HSW	SPP	SYP
**Variance**				
**Genotype (G)**	320.8***	24.8***	1,144.5***	11.9***
**Environment (E)**	386.1***	1.3***	861.8***	22.9***
**G × E**	73.8***	1.7***	329.4***	5.8***
**Error**	259.5	2.2	919.5	17.7
**Broad-sense heritability**	0.85	0.99	0.89	0.80

***represents the significance level at *p< 0.0001*.

**FIGURE 1:**
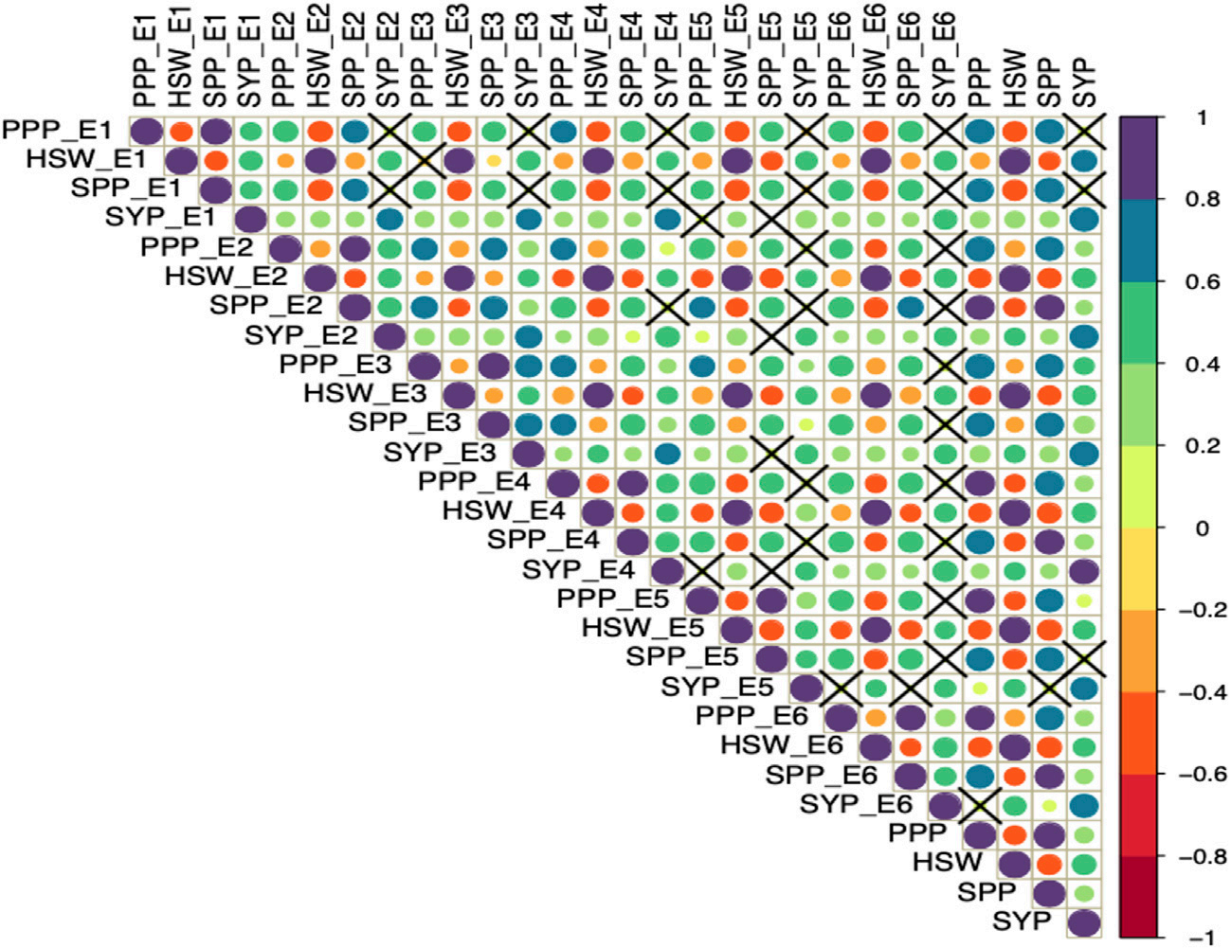
Pearson correlation analysis of yield and yield-related traits evaluated across diverse environments. The four traits including seed yield per plant (SYP), number of pods per plant (PPP), number of seeds per plant (SPP), and 100-seed weight in grams (HSW) were evaluated in six environments (E1, E2, E3, E4, E5, and E6) and the combined environment. The color and size of the circle reflect the strength of correlation. The non-significant correlations (*p* > 0.05) are indicated with a cross in individual cells.

Furthermore, Pearson correlation analysis revealed that SYP has a positive significant correlation with PPP, SPP and HSW (Figure 1). Whereas, HSW was negatively correlated with PPP and SPP in all the studied environments. Also, PPP showed a positive correlation with SPP across all six environments.

### Marker quality control, population structure, and linkage disequilibrium

**FIGURE 2:**
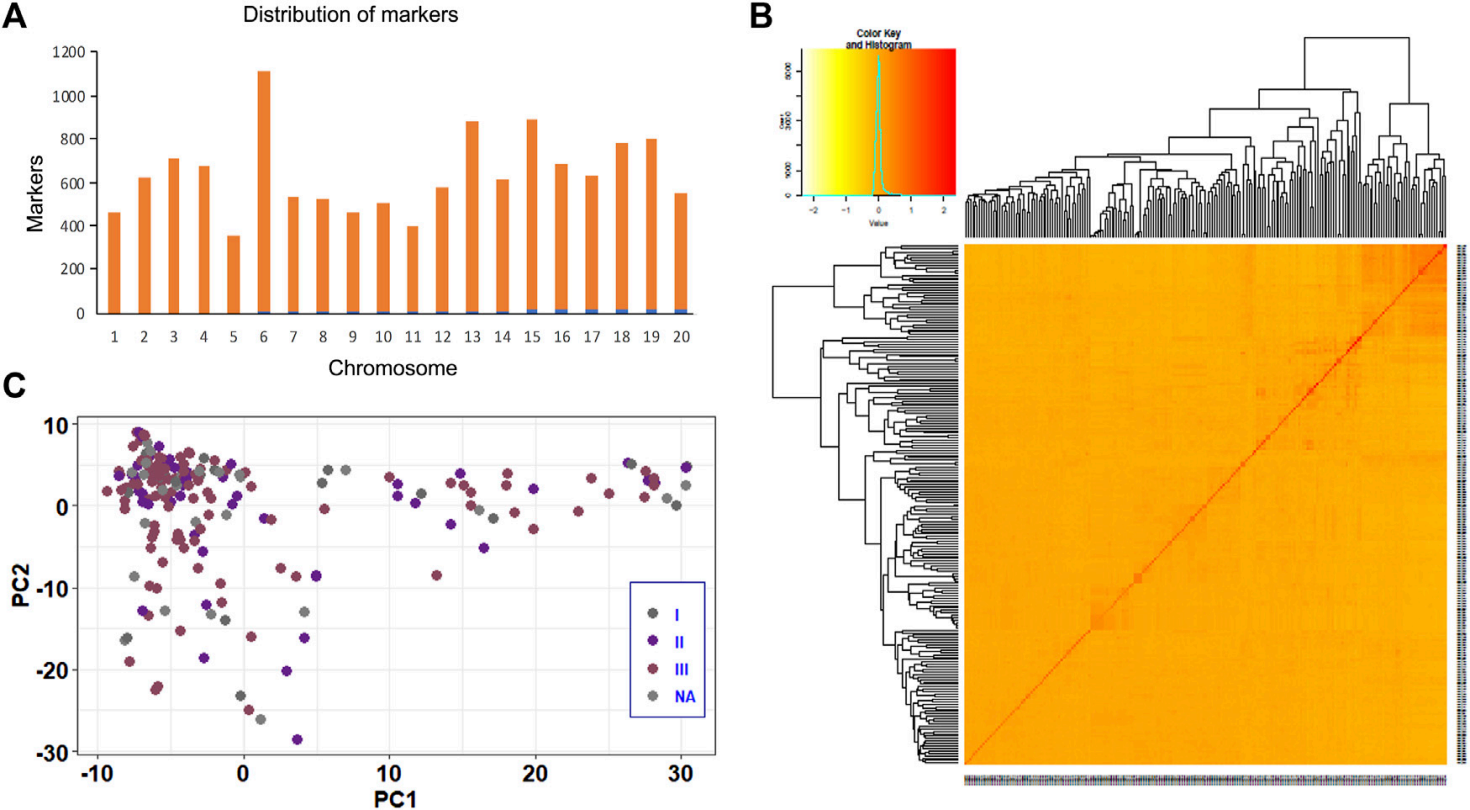
Kinship plot and population structure analysis of the soybean panel using 12,617 SNP markers. **(A)** Distribution of 12,617 SNP markers across 20 soybean chromosomes used for GWAS and GP analysis. **(B)** Kinship plot depicting the relationship among 211 soybean genotypes. **(C)** Population structure analysis of 211 soybean genotypes.

The quality control analysis retained 12,617 SNPs across 211 soybean genotypes at a genotyping rate of 99% after removing SNPs that failed the missingness, minor allele frequency, and Hardy-Weinberg exact tests. The markers were distributed across the soybean genome, with the highest (1,112) and lowest (352) number of markers present on Chr.06 and Chr.05, respectively (Figure 2A). Heatmaps and dendrograms of the kinship matrix, based on 12,617 polymorphic SNPs for the studied genotypes, indicated that there was no clear clustering among the genotypes (Figure 2B). The population structure based on soybean ecological regions in China also revealed a continuous distribution without any distinct structure (Figure 2C).

**FIGURE 3:**
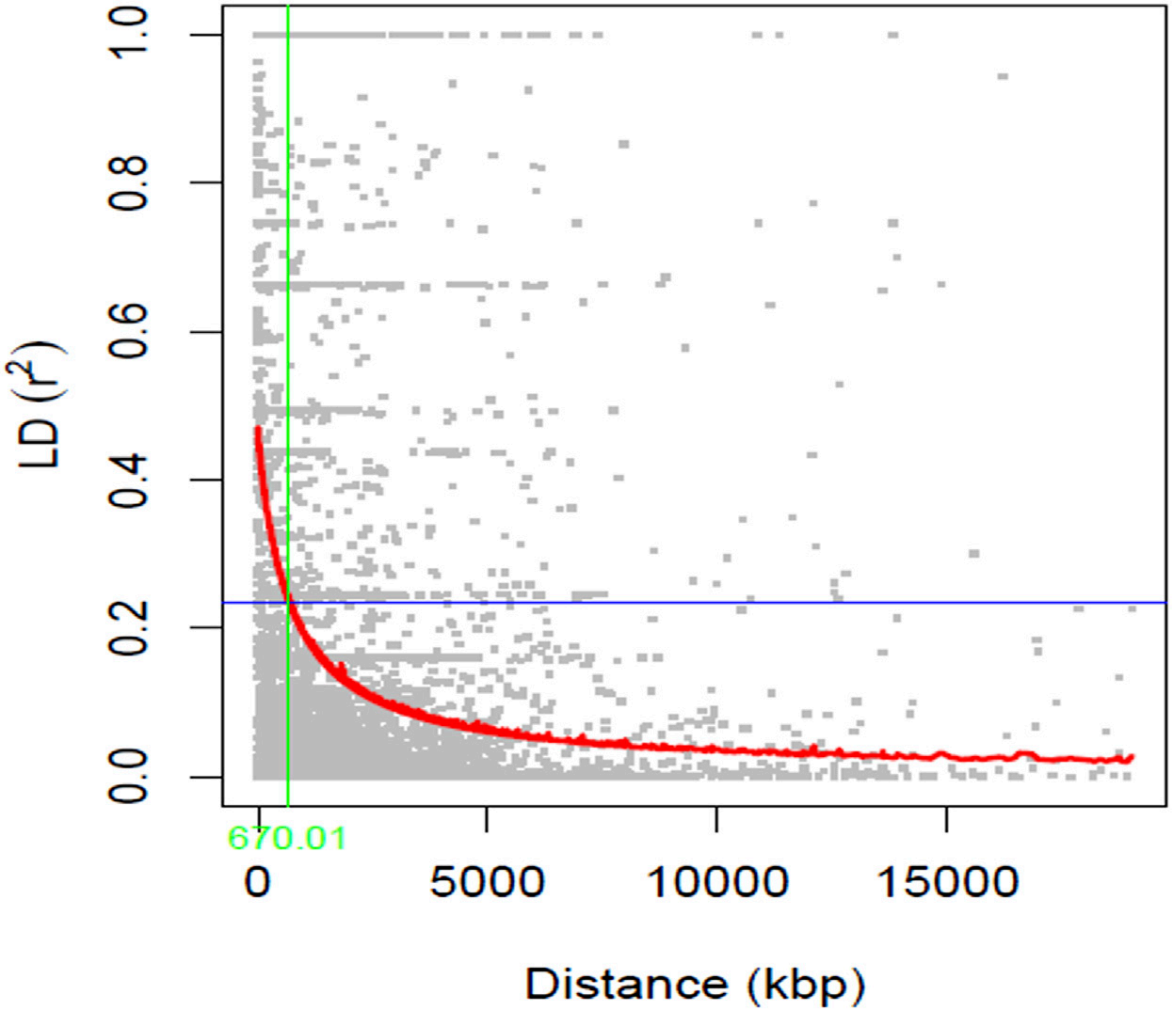
A scatter plot (*r* ^*2*^ values) of pairwise SNPs showing genome-wide linkage disequilibrium (LD) decay. The red curve line represents the smoothing spline regression model fitted to LD decay. The vertical green line indicates the genetic distance (670 Kbp) at which the LD half-decay (*r* ^*2*^ = 0.24, the horizontal blue line) intersect with the LD decay curve.

The graphical representation of the linkage disequilibrium characteristics of the 211 soybean genotypes is presented in Figure 3. The average *r* ^2^ value of the genome was 0.12, and the LD decay was found to initiate at an *r* ^2^ value of 0.47 and reached half-decay at 0.24. The LD decay curve intersected with the half-decay at 670 Kbp, which represents the genome-wide critical distance to detect linkage. Hence, markers associated with the same trait within this distance were considered as a single QTL.

### Marker-trait associations for yield-related traits

A total of 57 SNPs detected using five different GWAS models were found to possess significant association with four studied traits across six different environments (Supplementary Figure S1; Table 2). These SNPs were distributed across 17 soybean chromosomes. Among the significant SNPs identified, the highest number of SNPs (10) are found on Chr.15, followed by eight and five SNPs on Chr.20 and Chr.11, respectively. Four significant SNPs were found each on Chr.04, Chr.06, and Chr.13; whereas, Chr.08 and Chr.12 possessed three significant SNPs each. The remaining ten chromosomes possessed one or two significant SNPs. Further, some of these SNPs were consistently detected in multiple environments, using different GWAS models, and were found to be associated with more than one studied trait. Such SNPs were considered stable MTAs. For example, the significant SNP (AX-93793,210) on Chr.11 was identified consistently in five individual environments (E1, E2, E3, E4, and E5) and the combined environment. In addition, this SNP was also identified through four different GWAS models (BLINK, FarmCPU, GLM, and MLMM), and was found to be associated with two yield-related traits (HSW and SPP). Similarly, another SNP (AX-93807,406) detected on Chr.13 was found to be significantly associated with HSW and SPP across five individual environments (E1, E2, E4, E5, and E6) and the combined environment. This SNP was also identified using three GWAS models (BLINK, FarmCPU, and GLM).

**TABLE 2:** Significant marker-trait associations identified for four yield and yield-related traits across six environments (E1, E2, E3, E4, E5, and E6) and a combined environment (COM) using five GWAS models.

Sr. No	SPP	Trait	Chr^a^	Pos. (bp)^b^	Environments	Model	*p*-value	MAF
1	AX-93668,616	SYP	1	11,556,172	E3 and COM	BLINK, FarmCPU, GLM	2.68E-06 to 6.87E-14	0.06
2	AX-93973,672	PPP	1	55,502,450	E1	BLINK, GLM	1.69E-08 to 7.65E-08	0.01
3	AX-93991,698	SYP	3	24,910,743	COM	BLINK, FarmCPU	3.23E-07 to 7.38E-07	0.04
4	AX-93703,924	PPP, SPP	4	4,291,705	E3, E6 and COM	BLINK, FarmCPU, GLM	1.33E-06 to 8.45E-07	0.04
5	AX-93707,240	HSW	4	1,611,547	E1 and E5	BLINK, FarmCPU, GLM	1.05E-12 to 9.43E-07	0.17
6	AX-94000,527	PPP	4	5,416,455	E2 and COM	FarmCPU, GLM	1.26E-06 to 4.17E-10	0.35
7	AX-94006,694	HSW	4	39,016,845	E3	BLINK, FarmCPU	5.37E-07 to 9.88E-07	0.45
8	AX-93922099	HSW	5	36,599,702	E1, E5 and COM	BLINK, FarmCPU	2.12E-09 to 8.33E-08	0.09
9	AX-93715,038	HSW	5	2,491,676	E1 and E4	FarmCPU, GLM	1.01E-07 to 6.23E-07	0.09
10	AX-93725,825	PPP	6	5,623,054	E1	BLINK, GLM	3.09E-07 to 3.63E-07	0.13
11	AX-93730,411	HSW	6	19,462,776	E2	BLINK, GLM	1.35E-09 to 2.78E-08	0.09
12	AX-93735,201	HSW	6	43,399,273	E4	BLINK, FarmCPU	1.07E-06 to 1.83E-06	0.14
13	AX-94033,285	SYP	6	48,786,905	E3	BLINK, FarmCPU	6.83E-09 to 9.40E-10	0.48
14	AX-94034,566	HSW, SYP	7	1,626,042	E3 and E3	BLINK, FarmCPU, GLM	1.24E-06 to 2.98E-07	0.32
15	AX-94050,700	SYP	8	15,859,520	COM	BLINK, FarmCPU	5.66E-10 to 9.74E-09	0.04
16	AX-94283,862	PPP	8	19,011,459	COM	BLINK, FarmCPU	1.27E-06 to 7.72E-08	0.04
17	AX-93755,601	PPP	8	19,248,053	E3	BLINK, FarmCPU	1.57E-10 to 5.25E-07	0.12
18	AX-93636,437	SYP	9	6,958,542	E2	BLINK, GLM	6.90E-07 to 9.77E-12	0.05
19	AX-93772,794	SYP	9	4,4,392,036	COM	BLINK, GLM	1.36E-06 to 1.78E-06	0.13
20	AX-93793,210	HSW, SPP	11	29,587,057	E1, E2, E3, E4, E5 and COM	BLINK, FarmCPU, GLM and MLMM	1.13E-08 to 7.01E-11	0.26
21	AX-93792,964	HSW, PPP	11	27,468,886	E5, E6	BLINK, FarmCPU, GLM	1.31E-07 to 5.42E-13	0.19
22	AX-94276,492	PPP, SPP	11	2,211,768	E3	BLINK, GLM	1.34E-08 to 8.75E-10	0.10
23	AX-93792,958	HSW	11	27,459,211	E2 and COM	BLINK, FarmCPU, GLM and MLMM	3.22E-06 to 8.67E-07	0.19
24	AX-94092,104	SYP	11	38,213,308	COM	BLINK, FarmCPU, GLM	1.98E-08 to 4.35E-07	0.09
25	AX-93797,890	PPP, SPP	12	5,802,813	E3 and E5	BLINK, FarmCPU	1.11E-06 to 9.02E-08	0.03
26	AX-93804,315	PPP	12	33,817,396	E1	FarmCPU, GLM	2.29E-08 to 3.72E-07	0.01
27	AX-93805,697	SYP	12	37,339,415	E4	BLINK, GLM	1.49E-07 to 3.29E-07	0.20
28	AX-93807,406	HSW, SPP	13	1,843,185	E1, E2, E4, E5, E6 and COM	BLINK, FarmCPU, GLM	1.17E-10 to 8.25E-08	0.28
29	AX-94104,132	HSW, SPP	13	1,704,148	E3 and COM	BLINK, FarmCPU, GLM	1.74E-06 to 9.50E-07	0.23
30	AX-94111,538	HSW	13	29,191,712	E5 and COM	BLINK, FarmCPU	1.00E-07 to 4.87E-07	0.03
31	AX-93810,993	HSW	13	13,752,815	E5	BLINK, FarmCPU	1.73E-06 to 4.86E-08	0.05
32	AX-94123,137	PPP, SPP	14	23,593,462	E3	BLINK, GLM	1.31E-10 to 9.43E-09	0.02
33	AX-94137,762	SYP	15	22,710,572	E5	BLINK, FarmCPU, CMLM, GLM and MLMM	1.64E-09 to 2.48E-06	0.04
34	AX-93648,081	HSW	15	1,479,982	E6	BLINK, FarmCPU, GLM	1.84E-07 to 6.39E-07	0.34
35	AX-94138,593	SYP	15	29,628,987	COM	BLINK, FarmCPU, GLM	2.98E-07 to 8.36E-15	0.01
36	AX-93647,998	PPP	15	14,306,514	COM	BLINK, GLM	1.10E-10 to 1.24E-06	0.05
37	AX-94139,057	SPP	15	32,570,960	E6	BLINK, GLM	1.07E-06 to 1.31E-07	0.07
38	AX-94139,404	SYP	15	37,194,980	E3	FarmCPU and MLMM	1.18E-13 to 2.51E-06	0.03
39	AX-94139,741	SPP	15	40,016,648	COM	BLINK, GLM	1.66E-07 to 4.89E-10	0.14
40	AX-94139,803	PPP	15	40,162,413	E2	BLINK, GLM	1.44E-08 to 6.40E-08	0.06
41	AX-93843,622	HSW	15	44,240,130	E5	FarmCPU, GLM	1.03E-06 to 4.93E-08	0.13
42	AX-93843,767	SPP	15	44,966,712	COM	BLINK, GLM	3.31E-06 to 9.03E-11	0.14
43	AX-93650,734	HSW	16	30,750,889	E3	BLINK, FarmCPU, GLM	2.98E-06 to 9.61E-09	0.13
44	AX-93855,303	SPP	16	33,390,841	E5	BLINK, FarmCPU, GLM	2.13E-09 to 7.13E-07	0.02
45	AX-93652076	SYP	17	1,3,931,777	E6	BLINK, FarmCPU, GLM	1.67E-06 to 4.45E-07	0.01
46	AX-93959,968	SPP	17	12,429,289	E2	FarmCPU, GLM	1.52E-06 to 4.87E-07	0.02
47	AX-94176727	HSW, PPP	18	46,137,043	E1, E2 and COM	BLINK, FarmCPU, GLM	1.22E-07 to 5.79E-07	0.04
48	AX-93869,048	PPP	18	3,396,703	E3	BLINK, FarmCPU	2.32E-06 to 3.28E-07	0.04
49	AX-93886,740	PPP	19	3,901,635	E3	FarmCPU, GLM	1.57E-07 to 3.77E-06	0.03
50	AX-94199992	PPP, SPP	20	12,095,298	E1, E3 and COM	BLINK, FarmCPU, GLM	1.27E-08 to 6.40E-10	0.05
51	AX-93901,622	SPP	20	10,355,416	E2 and E3	BLINK, FarmCPU, GLM and MLMM	1.88E-06- to 4.95E-07	0.01
52	AX-93903,055	PPP	20	17,413,084	E1 and E2	BLINK, FarmCPU, GLM	1.10E-07 to 8.65E-09	0.01
53	AX-94198582	PPP	20	4,715,203	E3	BLINK, GLM	2.07E-09 to 2.67E-13	0.08
54	AX-94201014	PPP	20	17,645,281	E3	GLM, MLMM	1.50E-06 to 8.73E-11	0.02
55	AX-93903,184	PPP	20	18,155,072	E3	BLINK, GLM	4.38E-11 to 7.33E-09	0.02
56	AX-94207999	HSW	20	42,330,677	E2	BLINK, GLM	6.29E-09 to 7.83E-07	0.30
57	AX-94292,257	PPP	20	45,876,916	E5	BLINK, FarmCPU	1.99E-07 to 2.36E-07	0.01

a Chromosome.

b Physical position; MAF (minor allele frequency).

Furthermore, three significant SNPs (AX-94199992, AX-93703,924, and AX-94176727) on Chr.20, Chr.04, and Chr.18, respectively, were consistently detected in the combined environment using three different models (BLINK, FarmCPU, and GLM) and showed association with two of the three traits viz., HSW, PPP, and SPP. The SNP (AX-93922099) was detected in the combined environment using two different models (BLINK and FarmCPU) and was associated with HSW. Few significant SNPs such as AX-93792,964, AX-94034,566, AX-93797,890, and AX-94104,132, present on Chr.11, Chr.07, Chr.12, and Chr.13, respectively, were found to be associated with two of the four studied traits, using up to three GWAS models in one or two individual environments and a combined environment. Moreover, eight SNPs (AX-93668,616, AX-93707,240, AX-93792,958, AX-94000,527, AX-93715,038, AX-94111,538, AX-93901,622, and AX-93903,055) were detected in one or two individual environment(s) and a combined environment. Each of these eight SNPs was associated with only one trait and identified using up to four different GWAS models. The SNPs (AX-94276,492 and AX-94123,137) were found to be associated with two traits (PPP and SPP) and were identified using two GWAS models (BLINK and GLM), but were identified in only one individual environment. The remaining 37 significant SNPs were found to be associated with only one trait and environment, and most of them were identified using two or three GWAS models.

Based on GWAS, we identified six significant SNPs (AX-93703,924, AX-93922099, AX-93793,210, AX-93807,406, AX-94176727, and AX-94199992) on Chr.4, Chr.5, Chr.11, Chr.13, Chr.18, and Chr.20, respectively, consistently in three or more than three environments and using multiple models (Tables 2, 3). Of these, five SNPs (AX-93807,406, AX-93793,210, AX-94199992, AX-93703,924, and AX-94176727) were found to be associated with two studied traits (Tables 2, 3). However, the SNP AX-93922099 was associated with only HSW (Tables 2, 3). Hence, based on the LD decay, the genomic regions (±670 kb) flanking these significant SNPs (AX-93807,406, AX-93793,210, AX-94199992, AX-93922099, AX-93703,924, and AX-94176727) were considered as QTLs viz., *qHSW-SPP13*, *qHSW-SPP11*, *qPPP-SPP20*, *qHSW5*, *qPPP-SPP4*, and *qHSW-PPP18*, respectively (Table 3). These QTLs/genomic regions represented stable genetic elements potentially regulating soybean yield-related traits.

**TABLE 3:** Stable QTLs/genomic regions identified for the studied traits in at least three or more environments.

QTL	Chr^a^	Rep.SPP^b^	Pos. (bp)^c^	Environments	Model	Related QTL	References
*qPPP-SPP4*	4	AX-93703,924	4,291,705	E3, E6 and COM	*BLINK, FarmCPU, GLM*	Novel QTL	Not available
*qHSW5*	5	AX-93922099	36,599,702	E1, E5 and COM	*BLINK, FarmCPU*	*Seed weight 34–9; Seed-yield 22–10*	Han et al., 2012; Du et al., 2009
*qHSW-SPP11*	11	AX-93793,210	29,587,057	E1, E2, E3, E4, E5 and COM	*BLINK, FarmCPU, GLM and MLMM*	*Seed weight 35–9*	Han et al. (2012)
*qHSW-SPP13*	13	AX-93807,406	1,843,185	E1, E2, E4, E5, E6 and COM	BLINK, FarmCPU, GLM	Novel QTL	Not available
*qHSW-PPP18*	18	AX-94176727	46,137,043	E1, E2 and COM	BLINK, FarmCPU, GLM	Novel QTL	Not available
*qPPP-SPP20*	20	AX-94199992	12,095,298	E1, E3 and COM	BLINK, FarmCPU, GLM	Novel QTL	Not available

a Chromosome.

b The representative SPP, with the min *p* value.

c Physical position.

The italic values indicate “QTL names” and “Gene IDs”.

### RNA-seq data revealed 15 putative genes regulating yield-related traits

Six QTLs/genomic regions were identified on Chr.04 (*qPPP-SPP4*), Chr.05 (*qHSW5*), Chr.11 (*qHSW-SPP11*), Chr.13 (*qHSW-SPP13*), Chr.18 (*qHSW-PPP18*), and Chr.20 (*qPPP-SPP20*) were further used to identify putative genes regulating yield-related traits in soybean. The gene models and their annotations underlying the physical intervals of these six QTL regions were downloaded from the SoyBase database to predict putative candidates (Supplementary Table S2). In total, 739 gene models were identified within the physical genomic interval of *qPPP-SPP4*, *qHSW5*, *qHSW-SPP11*, *qHSW-SPP13*, *qHSW-PPP18* and *qPPP-SPP2*. However, by considering gene annotation, we selected 113 gene models within their physical genomic interval, which consisted of 31, 33, 10, 11, 14, and 14 genes underlying *qPPP-SPP4*, *qHSW5*, *qHSW-SPP11*, *qHSW-SPP13*, *qHSW-PPP18* and *qPPP-SPP20*, respectively (Supplementary Table S2). In addition, RNA-seq data for samples collected across different stages of soybean growth and development (www.soybase.org) was downloaded and analyzed for identifying putative genes underlying the QTL intervals (Supplementary Table S3). The RNA-seq data of these genes are represented using a heatmap (Supplementary Figure S2). Based on the *in-silico* analysis of gene expression data and gene annotations, we predicted a total of 15 putative candidates underlying six QTLs. These include 6, 2, 2, 1, 3, and 1 gene underlying *qPPP-SPP4*, *qHSW5*, *qHSW-SPP11*, *qHSW-SPP13*, *qHSW-PPP18*, and *qPPP-SPP20*, respectively (Table 4).

**TABLE 4:** Putative genes underlying six QTLs and their gene annotation.

QTL name	Gene IDs	Chrom-osome	Gene functional annotation
*qPPP-SPP4*	*Glyma04g05500*	04	Protein folding; abiotic stress response; positive regulation of transcription
	*Glyma04g05520*	04	NA
	*Glyma04g05580*	04	Gluconeogenesis; glycolysis; translational initiation; abiotic stress response
	*Glyma04g05690*	04	Lipid biosynthetic process
	*Glyma04g05720*	04	Protein folding; abiotic stress response
	*Glyma04g05800*	04	Photosynthesis
*qHSW5*	*Glyma05g31250*	05	Acetyl-CoA metabolic process; abiotic stress response; polysaccharide transport; sterol biosynthetic process; brassinosteroid biosynthetic process
	*Glyma05g31260*	05	Nuclear division; cytokinesis by cell plate formation; chromatin silencing; nucleolus organization; biological process; cell proliferation; histone phosphorylation; histone H3-K9 methylation
*qHSW-SPP11*	*Glyma11g28990*	11	NA
	*Glyma11g29000*	11	Protein N-linked glycosylation; ethylene biosynthetic process; sugar mediated signaling pathway; stem cell division; proteasomal ubiquitin-dependent protein catabolic process; cotyledon development; regulation of post-embryonic root development
*qHSW-SPP13*	*Glyma13g01950*	13	Carbohydrate metabolic process; regulation of meristem growth
*qHSW-PPP18*	*Glyma18g38490*	18	Regulation of transcription; gibberellin biosynthetic process; response to auxin stimulus; response to abscisic acid stimulus; gibberellic acid mediated signaling pathway; embryo development; terpenoid biosynthetic process; cotyledon development; cell division
	*Glyma18g38570*	18	Cell morphogenesis; protein N-linked glycosylation; N-terminal protein myristoylation; cell growth; protein ubiquitination; regulation of protein localization; protein autophosphorylation; Golgi vesicle transport; root hair elongation
	*Glyma18g38610*	18	Regulation of transport
*qPPP-SPP20*	*Glyma20g08580*	20	Actin filament organization; regulation of stomatal movement; regulation of protein localization

The italic values indicate “QTL names” and “Gene IDs”.

### Allelic effects of stable marker-trait associations

**FIGURE 4:**
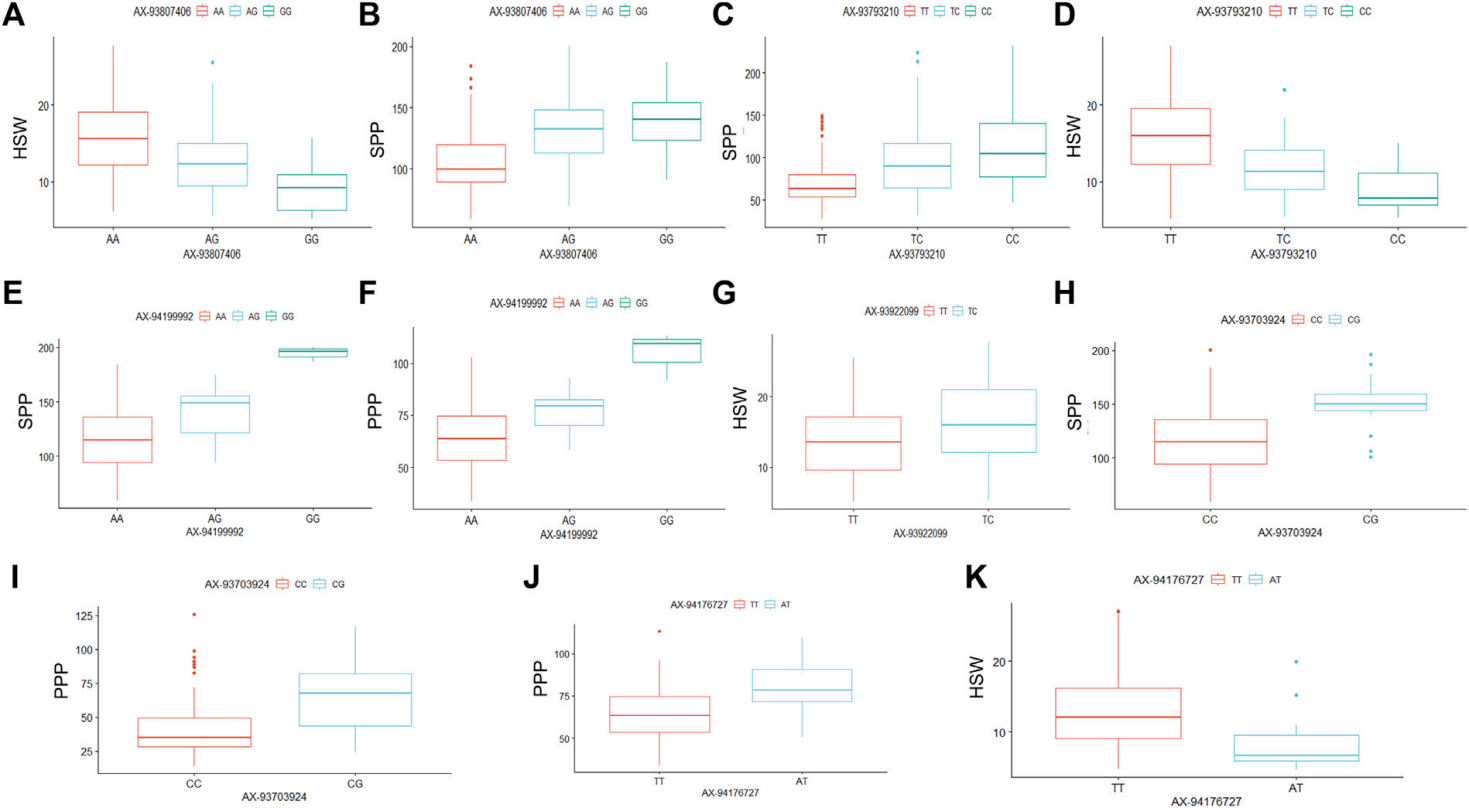
Allele-effect analysis for six stable significant SNPs including AX-93807,406 **(A,B)**, AX-93793,210 **(C,D)**, AX-94199992 **(E,F)**, AX-93922099 **(G)**, AX-93703,924 **(H,I)**, and AX-94176727 **(G,K)**. The box plot depicts the number of the alleles for each of the six significant SNPs in 211 soybean genotypes, and the contribution of these alleles to the phenotypic variation observed for yield and yield-related traits.

The six significant SNPs (AX-93703,924, AX-93922099, AX-93793,210, AX-93807,406, AX-94176727, and AX-94199992), showing stable MTAs with yield-related traits were further used to determine the effects of their individual alleles on the studied traits (Figure 4). The alleles of these six SNP markers showed substantial effects on yield-related traits combined from all the six environments (Figure 4). However, the number of alleles for each of these six SNP markers in the whole soybean population varied from two to three. For example, the SNP markers AX-93807,406, AX-93793,210, and AX-94199992 each possessed three different alleles; whereas, AX-93922099, AX-93703,924, and AX-94176727 possessed two alleles each (Figure 4). The SNP marker AX-93807,406 possessed three alleles (AX-93807406-AA, AX-93807406-AG, and AX-93807406-GG), and were found to regulate HSW and SPP (Figures 4A,B). The AX-93807406-AA, AX-93807406-AG, and AX-93807406-GG alleles governed higher, intermediate, and lower HSW, respectively; whereas these same alleles regulated lower, intermediate, and higher SPP, respectively. The SNP marker AX-93793,210 is associated with two yield-related traits (HSW and SPP), and all three alleles of this marker (AX-93793210-TT, AX-93793210-TC, and AX-93793210-CC) showed significantly different allelic effects on both HSW and SPP (Figures 4C,D). The allele AX-93793210-TT was associated with higher HSW, whereas AX-93793210-CC was associated with lower HSW. The effect of AX-93793210-TC on HSW was intermediate between that of AX-93793210-TT and AX-93793210-CC. However, the effect of three alleles of AX-93793,210 on SPP was found to be opposite to that of HSW, which also supports the negative correlation between HSW and SPP.

Moreover, three alleles of SNP marker AX-94199992, including AX-94199992-AA, AX-94199992-AG, and AX-94199992-GG, were found to govern three diverse phenotypes of SPP and PPP (Figures 4E,F). Besides, these three alleles of AX-94199992 regulated SPP and PPP phenotype in the same order as lower, intermediate and higher, respectively, which further supports the positive correlation of SPP and PPP traits. The two alleles of marker AX-93922099, including AX-93922099-TT and AX-93922099-TC, showed a significant difference in the regulation of HSW. For instance, the allele AX-93922099-TT governed lower HSW; whereas, the allele AX-93922099-TC was associated with higher HSW (Figure 4G). Similarly, the AX-93703,924 marker governed SPP and PPP traits, and the two alleles of this marker (AX-93703924-CC and AX-93703924-CG) regulated lower and higher trait values, respectively, for both the traits (Figures 4H,I). The SNP marker AX-94176727, possessing two alleles (AX-94176727-TT and AX-94176727-AT), regulated contrasting phenotypes of HSW and PPP (Figures 4J,K). For instance, AX-94176727-TT and AX-94176727-AT regulated lower and higher HSW, respectively; whereas, the same alleles governed higher and lower PPP values, respectively. These results are per the negative correlation observed between HSW and PPP.

### Haplotypes for yield-related traits

**FIGURE 5:**
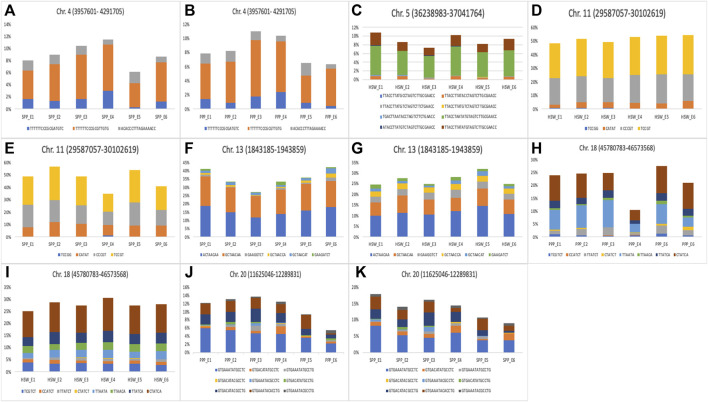
Haplotype analysis for yield and yield-related traits in soybean. **(A–K)** The bar plot represents haplotype alleles identified for haplotype block on **(A)** Chr.04 (3,957,601–4291,705 bp) **(B)** Chr.04 (3,957,601–4291,705 bp) **(C)** Chr.05 (36,238,983–3,7,041,764 bp) **(D)** Chr.11 (29,587,057–30102,619 bp) **(E)** Chr.11 (29,587,057–30102,619 bp) **(F)** Chr.13 (1,843,185–1943,859 bp) **(G)** Chr.13 (1,843,185–1943,859 bp) **(H)** Chr.18 (45,780,783–46,573,568 bp) **(I)** Chr.18 (45,780,783–46,573,568 bp) **(J)** Chr.20 (11,625,046–12,289,831 bp), and **(K)** Chr.20 (11,625,046–12,289,831 bp), and their contribution to the regulation of yield and yield-related traits. Six haplotype blocks were identified by considering six stable significant SNPs (AX-93807,406, AX-93793,210, AX-94199992, AX-93922099, AX-93703,924 & AX-94176727) as reference markers.

The six stable markers mentioned above were used as a reference for the identification of haplotypes for yield-related traits. These stable markers were located on Chr.04 (AX-93703,924), Chr.05 (AX-93922099), Chr.11 (AX-93793,210), Chr.13 (AX-93807,406), Chr.18 (AX-94176727), and Chr.20 (AX-94199992). All the markers that were in strong LD (within ±670 kbp) with these six SNP markers, represented a haplotype block/locus (Figure 5; Table S4). For example, 17 SNP markers were in strong LD with the reference marker AX-93703,924 (3,957,601–4291,705) and formed a haplotype block. Three haplotype alleles were identified within this haplotype block, in the soybean population (Figure 5A). These three haplotype alleles identified on Chr.04 showed significant differences in the phenotypes of SPP and PPP. Further, the reference marker AX-93922099 (36,238,983–3,7,041,764) formed a haplotype block with 26 markers, which consisted of eight haplotype alleles (Figure 5B). Substantial phenotypic variance in HSW was observed for haplotype alleles present within this haplotype block on Chr.05 (Figure 5C). The marker AX-93793,210 (29,587,057–30102,619) constituted a haplotype block with five SNP markers constituting four haplotype alleles. Variation in these four alleles led to significant phenotypic variation in HSW and SPP traits (Figures 5D,E).

Eight SNP markers were associated with AX-93807,406 (1,843,185–1943,859), which represented a haplotype block and constituted six haplotype alleles. All the six alleles identified within this haplotype block showed significant differences in the phenotypes of HSW and SPP (Figures 5F,G). Similarly, six SNPs were in association with the reference marker AX-94176727 (45,780,783–46,573,568), which together formed a haplotype block on Chr.18. Eight alleles identified within this haplotype block showed substantial variation in the phenotypes of HSW and PPP (Figures 5H,I). Further, 13 SNP markers were in close association with AX-94199992 (11,625,046–12,289,831) and formed a haplotype block representing nine haplotype alleles (Figures 5J,K). The haplotype alleles of the AX-94199992 (11,625,046–12,289,831) block showed significant phenotypic variation in PNP and SNP (Figures 5H,I). Although haplotype alleles for most haplotype blocks showed significant differences in the phenotypes of different yield-related traits in six individual environments, a few exceptions were also observed. For example, the haplotype alleles of AX-93922099 (36,238,983–3,7,041,764) block on Chr.05 did not show significant phenotypic variation in HSW for E3 (NT1) and E6 (YZ2). Moreover, the haplotype alleles of all other haplotype blocks showed a significant phenotypic difference (*p < 0.05*) in their associated traits across all six environments. The phenotypic variance contributed by the alleles of different haplotype blocks to the associated traits across six environments is shown in Figure 5. The list of markers that are in close association with the reference markers and the effects of the common haplotypes are provided in Supplementary Table S4.

### Genomic prediction

**FIGURE 6:**
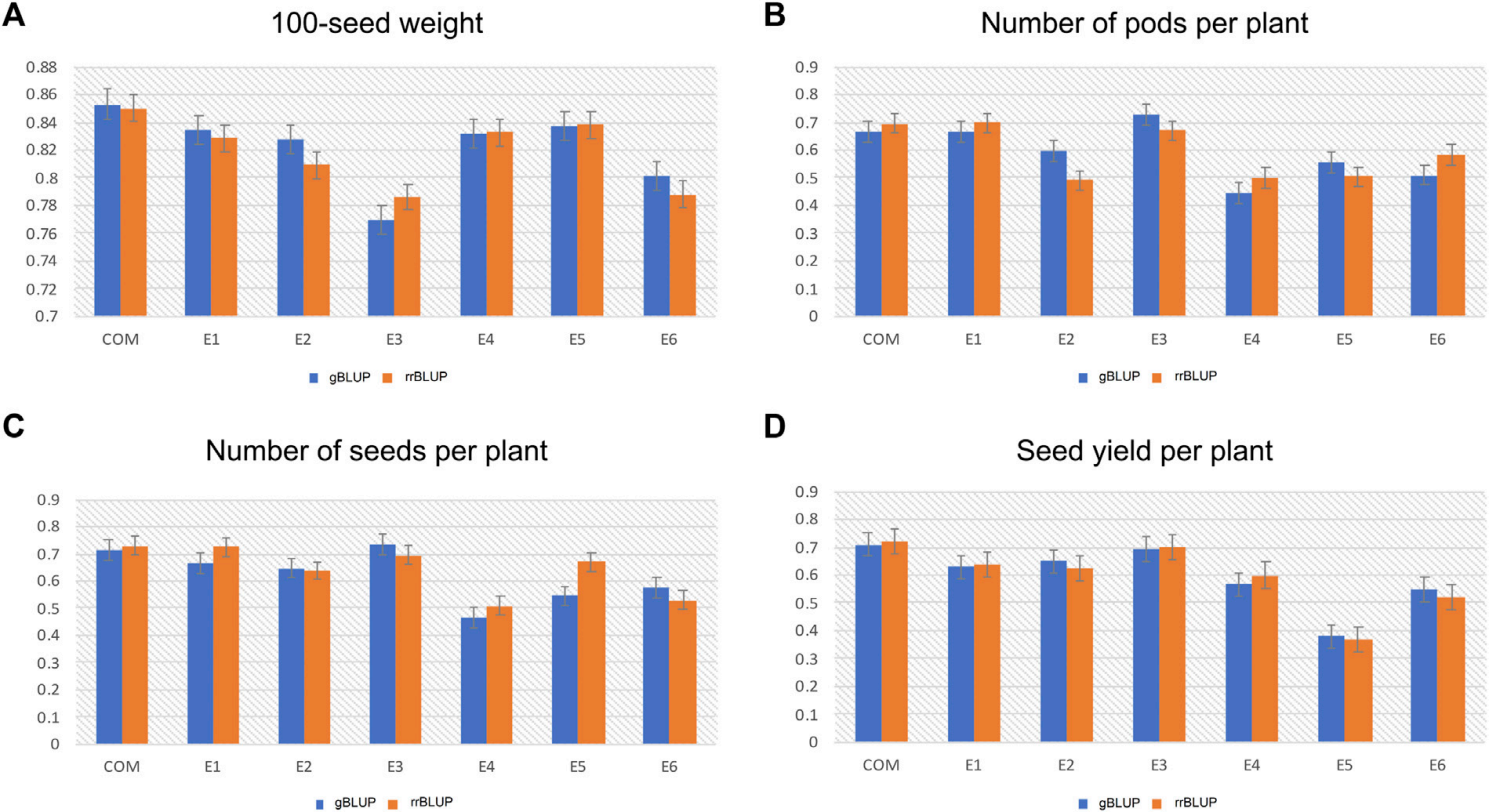
Histograms showing the genomic prediction accuracy of the genomic best linear unbiased prediction (gBLUP) and ridge regression best linear unbiased prediction (rrBLUP) models for **(A)** 100-seed weight (HSW) **(B)** Number of pods per plant (PPP) **(C)** Number of seeds per plant (SPP), and **(D)** Seed yield per plant (SYP), across six different environments (E1, E2, E3, E4, E5, and E6).

The genome-wide prediction accuracy values obtained from the gBLUP and rrBLUP approaches for the studied yield-related traits are presented in Figure 6. Based on the gBLUP approach, the GP accuracy of HSW among different environments ranged between 0.76 and 0.85 (Figure 6A). The E3 environment showed the lowest GP accuracy (0.76), while the combined environment displayed the highest GP accuracy (0.85) for HSW (Figure 6A). For the PPP trait, the E4 environment had the lowest GP accuracy with 0.44, while the highest accuracy was recorded in the E3 environment (0.72) (Figure 6B). Moreover, the GP accuracy for SPP varied from 0.46 to 0.72 in E4 and E3 environments, respectively (Figure 6C). Similarly, in the case of SYP, the GP accuracy was found to be highest (0.70) in the combined environment, whereas the lowest GP accuracy of 0.37 was observed in the E5 environment (Figure 6D). A similar trend in the genome-wide prediction accuracy was observed using the rrBLUP approach: HSW ranged between 0.78 and 0.85 for E3 and combined environment, respectively (Figure 6A); and PPP varied from 0.49 to 0.69. The PPP trait possessed the highest GP accuracy in the E1 environment and the lowest accuracy in the E2 environment (Figure 6B). Also, the GP accuracy for SPP and SYP respectively ranged from 0.50 (E4) to 0.73 (combined environment) and 0.36 (E5) to 0.72 (combined environment) (Figures 6C,D).

## Discussion

Yield-related traits are important characters associated with yield and directly govern the productivity and quality of crops (Hina et al., 2020). They also represent selection targets in plant breeding programs when direct selection for yield is complex. Hence, crop germplasm collections are characterized for yield-related traits to facilitate crop improvement (Adeboye K. A. et al., 2021). In soybean, a complex inheritance pattern of yield and its sensitivity to the environment have been documented (Lee et al., 2015). Therefore, it has remained the long-term goal of breeders to improve soybean yield by manipulating yield-related traits. Determining the genetic basis of yield-related traits is a key step in soybean improvement strategy for developing varieties with higher yield potential.

In the present study, 211 soybean germplasm accessions were characterized in six field trials for four yield-related traits. The genotypic performance of the soybean germplasm based on all these traits varied significantly, indicating the possibility of genetic improvement. Moreover, the medium to high broad-sense heritability observed for these traits across environments is an indication that the same phenotypic performance is achievable if grown in the same environment. However, the significant environmental variations observed for all the four yield-related traits studied suggest possible complexity in their inheritance pattern which may lead to difficulty in breeding efforts. These findings corroborate many earlier reports on these yield-related traits in soybean germplasm (Hu et al., 2014; Diers et al., 2018; Klein et al., 2020; Li et al., 2020). Moreover, correlation analysis revealed a positive association of SYP with the three yield-related traits (PPP, SPP, and HSW) consistent with the findings of Malik et al. (2006). The HSW was negatively correlated with PPP and SPP; while PPP was positively associated with SPP. These results are in accordance with previous reports (Malik et al., 2006; Liu et al., 2011; Li et al., 2020).

Based on the high genetic variability observed, the studied yield-related traits were subjected to further analysis to unravel their genetic basis and pave the way for their improvement through marker-assisted breeding (MAB). MAB involves the identification of genetic markers that are associated with the trait of interest in a defined germplasm collection, such as bi-parental population or a panel of diverse accessions as used in this study. Marker-trait association using a panel of diverse accessions is often confounded by several factors, including genotyping error, population structure, and linkage disequilibrium. These factors are responsible for the high rate of false associations that are not useful in MAB. In this study, the discovery of false association was reduced by ensuring only quality markers which included a total of 12.617 SNPs at genotyping rate of 99%, which were retained for further analysis. Although the population structure of the germplasm collection used in this study appears continuous with no definite stratification, the LD decay distance of 670 kb within which significant association may be defined as relatively large. In self-pollinating plants such as soybean, LD may range over several hundred Kbp leading to the inclusion of many candidate genes in a single LD block exhibiting a significant signal (Gupta et al., 2005; Yano et al., 2016). Moreover, several studies have revealed that the discovery of false positives arising from population structure in crops may not be completely controlled (Myles et al., 2009; Hamblin et al., 2011; Lipka et al., 2015). Based on this, we explored various statistically robust models for the genome-wide marker-trait association analysis.

Hundreds of QTLs for yield and yield-related traits have been reported in soybean mainly by using the low-resolution approach of linkage mapping. Rarely any of these QTLs have been used for breeding high-yielding soybean varieties (Karikari et al., 2019). In this context, GWAS facilitates enhanced resolution and accuracy for mining genetic loci for four yield-related traits (Assefa et al., 2019; Zhang W. et al., 2021). In the current study, we report a total of 57 MTAs associated with four traits. These MTAs were detected on all chromosomes of soybean, except Chr.02 and Chr.10, indicating the complex genetic regulation of soybean yield, which is in agreement with previous reports (Li et al., 2019; Hu et al., 2020). Many significant MTAs were detected in one environment and some in five environments, suggesting the presence of environmental influence on these traits. Per the present results, the interaction of QTLs with the environment has been previously documented (Fang et al., 2020; Hu et al., 2020).

Significant SNPs reported in more than three environments and using different models were considered stable MTAs. The regions within ±670 kb flanking six significant SNPs were referred to as stable QTLs, based on LD decay (Wang et al., 2016). The QTL on Chr.11 associated with HSW has been previously reported in the genomic region between 27,790,963–32,194,459 bp (Han et al., 2012), and the genomic region underlying *qHSW-SPP11* was found to co-locate with the same physical interval. Therefore, *qHSW-SPP11* might be similar to *Seed weight 35–9*, as reported earlier by Han et al. (2012). Notably, compared to *Seed weight 35–9*, the physical interval of *qHSW-SPP11* has been considerably decreased in the present study. Furthermore, *qHSW5* identified in the present study was found to co-locate with two previously identified QTLs viz., Seed weight 34–9 (8,665,543–40,414,305 bp) and Seed-yield 22–10 (35,536,817–37,612,231 bp) on Chr.05 (Kraakman et al., 2004; Du et al., 2009; Han et al., 2012). However, the remaining four QTLs (*qHSW-SPP13*, *qPPP-SNP20*, *qPPP-SPP4*, and *qHSW-PPP18*) identified in the current study represented novel QTLs. The physical intervals of *qHSW-SPP11* and *qHSW5* were considerably reduced in the present study. This ability of the GWAS allows for its increased utility in crop breeding programs for developing high-yielding stress-tolerant soybean varieties (Zargar et al., 2015; Yu et al., 2019).

The favourable and unfavourable alleles can be easily determined either with or without considering the heterozygous SNPs in plant species (Soltani et al., 2017; Su et al., 2019). For instance, Wu et al. (2016) set the heterozygous SNPs as missing and only used the homozygous SNPs for a GWA study in *Brassica napus*. Soybean is a highly heterozygous crop species with a complex background, in which the presence of heterozygous loci is very common. A locus is considered to be in a heterozygous state if the depth of the minor allele is larger than one-third of the total sample depth during SNP calling (Chong et al., 2016). The above-mentioned six stable significant SNP loci (AX-93807,406, AX-93793,210, AX-94199992, AX-93922099, AX-93703,924, and AX-94176727) associated with yield-related traits were not all in a heterozygous state in the GWAS population. For example, the SNP markers AX-93807,406, AX-93793,210, and AX-94199992 were heterozygous, whereas the remaining three markers AX-93922099, AX-93703,924, and AX-94176727 were homozygous.

Trait values governed by the heterozygous alleles of three SNP markers (AX-93807,406, AX-93793,210, and AX-94199992) were intermediate between two homozygous alleles which control the extreme phenotypes of yield-related traits. However, two homozygous alleles identified for the remaining three SNP markers (AX-93922099, AX-93703,924, and AX-94176727) regulate contrasting/extreme trait values of the corresponding traits. As a result, the SNP alleles with higher trait effect, i.e., which increase the target trait, were defined as “favourable alleles”; whereas, SNP alleles regulating the lowest trait value were defined as “unfavourable alleles”. However, the heterozygous alleles possessing trait effect between favourable and unfavourable alleles were referred to as “intermediate alleles”. This classification assisted in the use of these alleles for yield modulation in soybean. To date, the effectiveness of marker-based gene pyramiding strategies in soybean has been demonstrated for soybean mosaic virus (Wang D. G. et al., 2017), Phytophthora rot and powdery mildew resistance (Ramalingam et al., 2020), and rust resistance (Yamanaka and Hossain, 2019). Hence, the elite alleles identified for yield-related traits within six significant SNP markers can be effectively used for developing high-yielding soybean varieties through MAB efforts. Negligible efforts have been made toward mining candidate genes for yield-related traits in soybean (Karikari et al., 2019; Li et al., 2019; Qi et al., 2020); except for two genes that have been reported viz., in (Jeong et al., 2012) and *PP2C-1* (Lu et al., 2011). In the present study, we predicted some putative genes underlying six QTLs identified based on the gene expression data and annotations. We selected only those genes whose functions were directly or indirectly related to regulating the seed yield of soybeans, such as seed oil, seed protein, photosynthesis, cell division or elongation, and phytohormones. The putative genes identified in the present study need further functional validation for their deployment in soybean breeding programs.

Recent studies have documented desirable haplotype alleles for important traits such as salinity tolerance in soybean (Patil et al., 2016), grain quality traits in rice (Wang X. et al., 2017), and drought stress tolerance in pigeon pea (Sinha et al., 2020). In the present study, haplotypes were constructed by using six significant SNP markers as a reference. These six stable markers possessed multiple significant SNP markers within the LD range of 670 kbp. Our results revealed that haplotype alleles identified within the haplotype blocks/loci regulated a diverse range of phenotypes of yield-related traits in soybean. The haplotype analysis revealed that, compared to individual SNP markers, the haplotype-based markers possessed a considerably higher number of alleles regulating a diverse range of phenotypic variation for the trait of interest, similar to previous studies (Zaitlen et al., 2005; Qian et al., 2017). Hence, haplotype-based markers provided more options to modulate the desired yield potential in soybean (Meuwissen et al., 2001). In the case of significant SNP markers identified, we found a maximum of three alleles in the GWAS population, which allowed to modify the yield of soybean at three levels. The incorporation of these haplotype alleles in soybean breeding programs can effectively improve yield potential in soybean. We propose that the haplotype-based breeding approach will assist in the selection of desirable plant genotypes possessing superior haplotype alleles (Varshney et al., 2019). Parent accessions with diverse haplotypes can be used for the generation of novel superior haplotypes. However, it is important to identify the interactive effects of diverse haplotypes of various genes regulating the trait of interest.

Genomic Prediction (GP) is a modern breeding approach that involves the use of genome-wide markers to estimate the breeding value of the genotypes at the genomic level (Meuwissen et al., 2001; Varshney et al., 2014). For the past 2 decades, GP has emerged as a powerful tool to select favourable genetic material for traits of interest (Bhat et al., 2016; Crossa et al., 2017). In the present study, GWAS identified minor-to-moderate effect QTLs. Thus, it was hypothesized that the GP method could be more appropriate to select high-yielding genotypes based on the overall marker effect (Varshney et al., 2014). Different statistical models have been developed and used for GP analysis (Daetwyler et al., 2010; Wang et al., 2018; Merrick and Carter, 2021). However, these methods mainly differ in the assumptions of the distribution and variances of marker effects (Wang et al., 2015). In the present study, we explored two approaches, including the gBLUP and rrBLUP, both of which are based on the mixed linear model statistical functions. Therefore, our results show a similar trend in their prediction accuracy as expected, suggesting their equal potential and efficiency in the prediction of yield-related traits in soybean.

The range of moderate to high GP accuracy of 100-seed weight and seed yield observed in our study is similar to the observation of Ravelombola et al. (2021) and Matei et al. (2018) in soybean based on rrBLUP approach. Similarly, Duhnen et al. (2017) reported a moderate GP accuracy for the seed yield of soybean based on the gBLUP approach. Moreover, moderate to high GP accuracy has been reported for yield-related traits from both rrBLUP and gBLUP approaches in other crops such as wheat (Ali et al., 2020), tea (Lubanga et al., 2021), rice (Xu et al., 2018), and chickpea (Roorkiwal et al., 2016). The genomic prediction results from our study revealed that accurate breeding values for the studied yield-related traits can be estimated at an earlier generation of soybean, which allows for yield improvement within a short breeding cycle.

## Conclusion

The present study used GWAS, haplotype analysis, and GP for studying the genetic architecture of four yield and yield-related traits in soybean. GWAS identified a total of 57 significant SNPs and six stable QTL regions (*qPPP-SPP4*, *qHSW5*, *qHSW-SPP11*, *qHSW-SPP13*, *qHSW-PPP18*, and *qPPP-SPP20*). Among these six QTLs, four QTLs (*qPPP-SPP4*, *qHSW-SPP13*, *qHSW-PPP18*, and *qPPP-SPP20*) were novel; whereas, the remaining two QTLs (*qHSW5* and *qHSW-SPP11*) were reported previously. Besides, a total of 15 genes underlying these six QTLs were prioritized as putative candidates. Allele-effect analysis of the six significant SNPs showed the presence of two or three alleles within each of these SNPs that regulated contrasting phenotypes of the associated traits. Moreover, multiple haplotype alleles detected within each of the six haplotype blocks regulated a diverse range of phenotypic variation for yield and yield-related traits. The GP analysis for four studied traits showed moderate to high accuracy using two methods (gBLUP and rrBLUP). The stable QTLs as well as the desirable SNP alleles and haplotype alleles (underlying these stable QTLs) identified for the yield-related traits can serve as potential resource for the improvement of soybean yield. After proper validation of these QTLs and alleles/haplotypes in different genetic backgrounds of soybean, they can be introduced into marker-assisted breeding programs for developing high-yielding soybean varieties. Besides, the putative candidate genes underlying these stable QTLs, after proper functional validation using overexpression or gene knockout studies, can be deployed in the development of high-yielding soybean varieties. Our study provided critical analyses of cultivated soybean genetic resources and identified novel genomic resources (QTLs and haplotype alleles) for soybean yield improvement programs.

## Data Availability

The original contributions presented in the study are publicly available. This data can be found here: “https://www.soybase.org/projects/SoyBase.C2021.03.php”.
