# Supplementary material for: Genome-wide association study, haplotype analysis, and genomic prediction reveal the genetic basis of yield-related traits in soybean (Glycine max L.)
Source: Front Genet. 2022 Aug 17;13:953833. doi: 10.3389/fgene.2022.953833 (PMC9677453; doi:10.3389/fgene.2022.953833)
Supplement: Supplementary file 1 [file DataSheet2.PDF]

Genome-wide association study, haplotype analysis, and genomic prediction reveal the genetic basis of yield and yield-related traits in soybean (*Glycine max* L.)

Javaid Akhter Bhat[1,2†*], Kehinde Adewole Adeboye[3†], Showkat Ahmad Ganie[4], Rutwik Barmukh[5], Dezhou Hu[1], Rajeev K. Varshney[5,6*], Deyue Yu[1*]

[1]Soybean Research Institution, National Center for Soybean Improvement, State Key Laboratory of Crop Genetics and Germplasm Enhancement, Nanjing Agricultural University, Nanjing 210095, China

[2]International Genome Center, Jiangsu University, Zhenjiang-212013, China

[3]Department of Agricultural Technology, Ekiti State Polytechnic, Isan 371106, Nigeria

[4]Plant Molecular Science and Centre of Systems and Synthetic Biology, Department of Biological Sciences, Royal Holloway University of London, Egham, Surrey, TW20 0EX, UK

[5]Center of Excellence in Genomics & Systems Biology, International Crops Research Institute for the Semi-Arid Tropics (ICRISAT), Hyderabad, 502324, India

[6]Murdoch's Centre for Crop & Food Innovation, State Agricultural Biotechnology Centre, Food Futures Institute, Murdoch University, Murdoch, Western Australia, Australia

[*]Correspondence:

javid.akhter69@gmail.com (Javaid Akhter Bhat);

rajeev.varshney@murdoch.edu.au (Rajeev K. Varshney);

dyyu@njau.edu.cn (Deyue Yu)

[†]Equal first author

Supplementary Figures

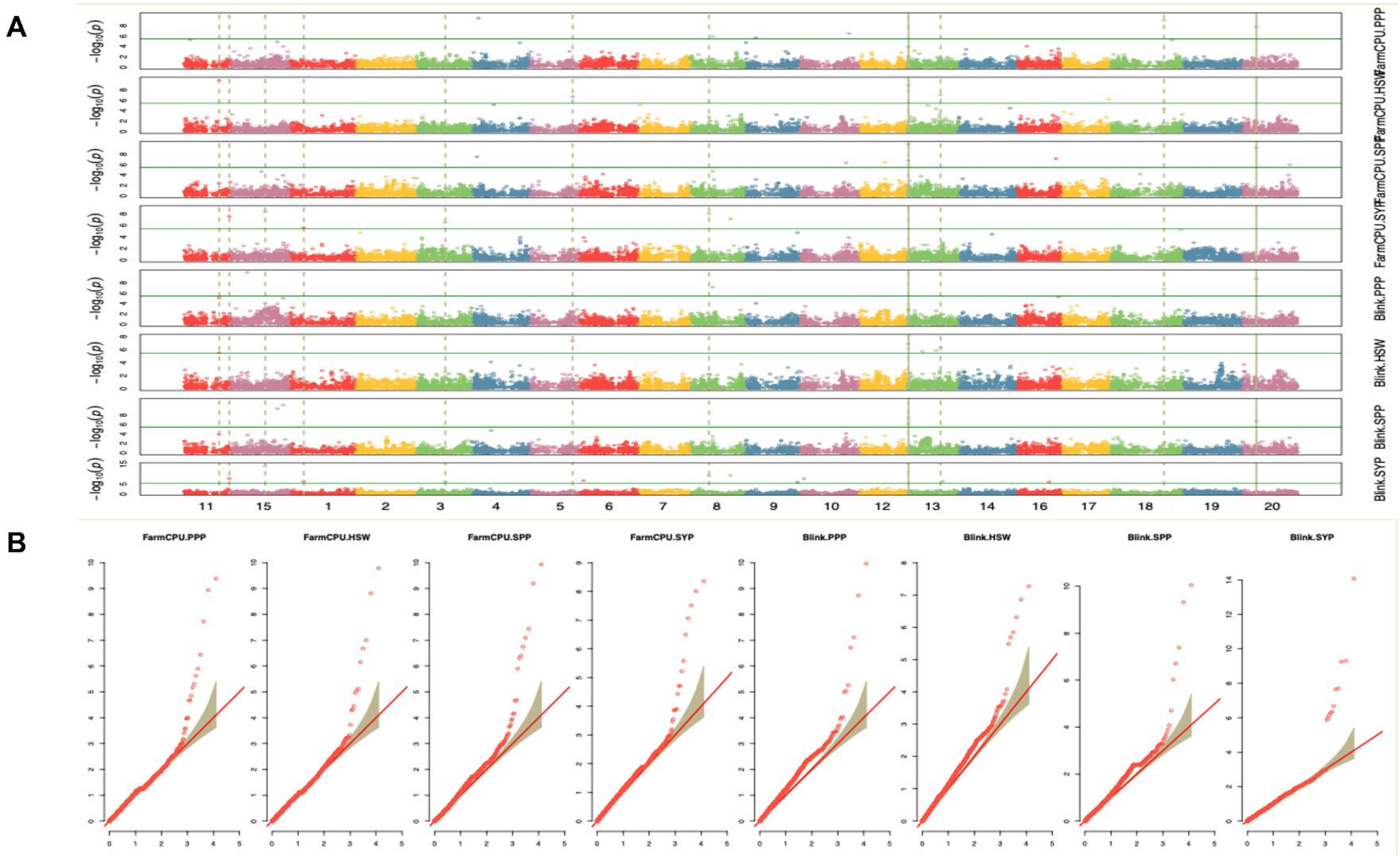

Supplementary Figure S1: Manhattan plot and Q-Q plot for traits with significant SNPs. (A) Manhattan plot for four traits viz., PPP, SPP, HSW, and SYP, identified using different GWAS models (FarmCPU and Blink). The green line indicates the significant threshold while the blue line shows the suggestive threshold. (B) Q-Q plot for GWAS results of four traits viz., PPP, SPP, HSW, and SYP, identified using different GWAS models (FarmCPU and Blink).

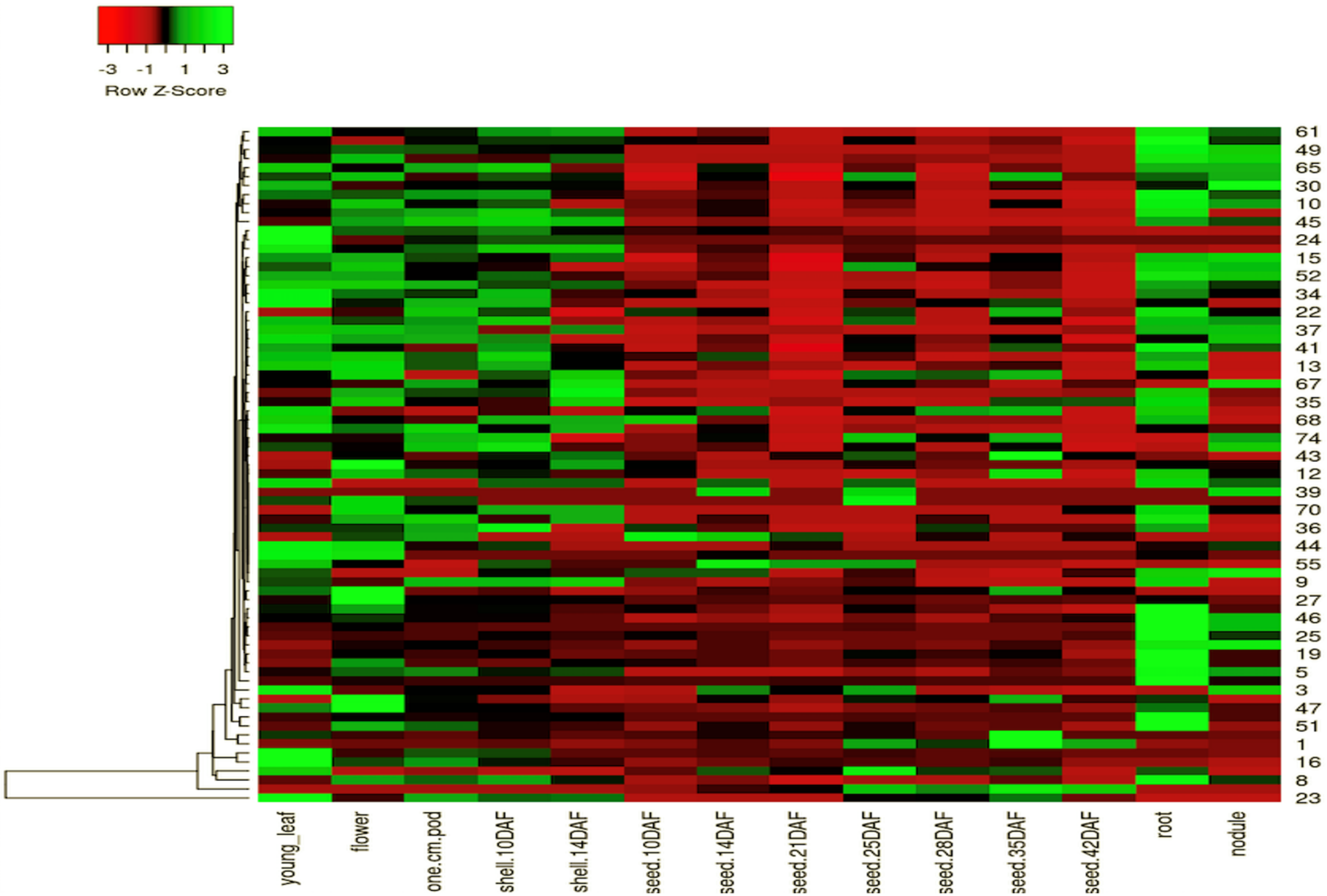

Supplementary Figure S2: Heat map showing the expression profiles of 75 candidate genes underlying six QTL regions (*qPPP-SPP4*, *qHSW5*, *qHSW-SPP11*, *qHSW-SPP13*, and *qHSW-PPP18*, and *qPPP-SPP20*), among different soybean tissues and development stages. Heat map was generated using the RNA-seq data downloaded from SoyBase database. Youngleaf—young leaf, One-cmpod—1 cm of pod, PS—pod shell, DAF—days after flowering, and S—seed.
